# Foraging optimally in social neuroscience: computations and methodological considerations

**DOI:** 10.1093/scan/nsaa037

**Published:** 2020-03-30

**Authors:** Anthony S Gabay, Matthew A J Apps

**Affiliations:** Department of Experimental Psychology, University of Oxford, Oxford OX1 2JD, UK; Wellcome Centre for Integrative Neuroimaging, University of Oxford, Oxford OX1 2JD, UK; Department of Experimental Psychology, University of Oxford, Oxford OX1 2JD, UK; Wellcome Centre for Integrative Neuroimaging, University of Oxford, Oxford OX1 2JD, UK; Christ Church College, Oxford OX1 1DP, UK

**Keywords:** computational neuroscience, social neuroscience, marginal value theorem, optimal foraging theory

## Abstract

Research in social neuroscience has increasingly begun to use the tools of computational neuroscience to better understand behaviour. Such approaches have proven fruitful for probing underlying neural mechanisms. However, little attention has been paid to how the structure of experimental tasks relates to real-world decisions, and the problems that brains have evolved to solve. To go significantly beyond current understanding, we must begin to use paradigms and mathematical models from behavioural ecology, which offer insights into the decisions animals must make successfully in order to survive. One highly influential theory—marginal value theorem (MVT)—precisely characterises and provides an optimal solution to a vital foraging decision that most species must make: the patch-leaving problem. Animals must decide when to leave collecting rewards in a current patch (location) and travel somewhere else. We propose that many questions posed in social neuroscience can be approached as patch-leaving problems. A richer understanding of the neural mechanisms underlying social behaviour will be obtained by using MVT. In this ‘tools of the trade’ article, we outline the patch-leaving problem, the computations of MVT and discuss the application to social neuroscience. Furthermore, we consider the practical challenges and offer solutions for designing paradigms probing patch leaving, both behaviourally and when using neuroimaging techniques.

## Introduction

How do we make decisions based on the value of different options during social interactions? This question has been at the core of social, computational and decision neuroscience in the last decade. To address it, research has largely focused on using approaches inspired by work in social psychology, from reinforcement learning (RL) and computer science, or from economics (O’Doherty *et al.*, 2015). These approaches have proven fruitful for better understanding many computations and the mechanisms guiding social behaviours (Behrens *et al.*, 2009; Ruff and Fehr, 2014; Apps *et al.*, 2016; Hill *et al.*, 2016; Lockwood *et al.*, 2016; FeldmanHall and Chang, 2018; Wittmann *et al.*, 2018). However, experimental designs and concepts can sometimes be somewhat artificial and may not reflect the kinds of decisions that humans and other animals regularly make in the real world. Recognition of this has led many to argue that we can get a better understanding of behaviours and their underlying neural mechanisms by turning to a different field: behavioural ecology (Kolling *et al.*, 2012; Pearson *et al.*, 2014; Kolling and Akam, 2017; Mobbs *et al.*, 2018).

The research in behavioural ecology aims to explain how the environment an animal is situated in shapes their decision-making behaviours. This work has demonstrated that animals are able to be almost optimal when making decisions that they would commonly face in their natural environment (Krebs *et al.*, 1974; Charnov, 1976; Mobbs *et al.*, 2018). Such optimality is not surprising: survival would have depended on the evolution of decision-making mechanisms that minimised predation risk or maximised reward intake when foraging. We contend that those same decision-making mechanisms may be at least partially conserved in humans and may be deployed when making a vast range of decisions, including during social interactions. Indeed, there is considerable evidence that there is overlap in the mechanisms that guide self-benefitting and other-oriented decisions, even if there are some dissociations (Zaki and Mitchell, 2011; Chang *et al.*, 2013; Wittmann *et al.*, 2018; Piva *et al.*, 2019). Thus, similar mechanisms that will be deployed when solving problems outlined within behavioural ecology may be applicable to social and non-social decisions alike.

New insights have already been found in behavioural and cognitive neuroscience by using paradigms inspired by models and frameworks from behavioural ecology, which may not have been discovered using the binary choice paradigms common in RL and neuroeconomics (Hayden *et al.*, 2011; Kolling *et al.*, 2012; Wolfe, 2013; Constantino and Daw, 2015; Lottem *et al.*, 2018; Le Heron *et al.*, 2019). As social behaviours may rely on the same mechanisms, new insights can be gained in social neuroscience by deploying frameworks from behavioural ecology. Although behavioural ecology has multiple branches, each of which may make different contributions to cognitive and social neuroscience, here we focus on one famous paradigm and a key theory in ecology: the patch-leaving problem. Marginal value theorem (MVT) (Charnov, 1976) provides a powerful optimal account of how animals should make patch-leaving decisions when foraging for rewards. In the patch-leaving problem, an agent must continually consider whether to stay in a location (patch) collecting rewards from a depleting resource, or travel elsewhere to find new ‘patches’ from which to harvest rewards.

We argue that humans make many social decisions that could be thought of as patch-leaving decisions, from partner choice, to decisions of when to join or leave a social group.

In this manuscript, we provide a ‘tools of the trade’ outline of MVT, its optimal computational model and the important considerations for designing a patch-leaving-based experiment. This will guide future studies that aim to use patch-leaving paradigms as fruitful, novel ways to probe the computational, cognitive and neural mechanisms underlying social decisions.

### The patch-leaving problem and optimal foraging

For many species, a vital problem that must be solved in order to survive, is how to forage for rewards (e.g. food) in patchy environments. Making ‘patch-leaving’ decisions sub-optimally can lead to an animal not having consumed enough energy to survive. What must animals consider in order to solve this problem? There are three key features that drive foraging decisions according to theories from behavioural ecology: the reward rate in a patch, the richness of the environment and the dispersion of patches.

When an animal arrives in a new location (a ‘patch’), typically it will be plentiful with a rewarding resource, so they will obtain rewards at a high rate. The more they collect, the more the resource is depleted, and the lower their foreground or instantaneous rate of reward becomes (e.g. the more apples you have collected from a tree, the less apples are left and the longer it takes to get each one). The total amount of reward in a patch and the rate at which a resource depletes are therefore vital for the animal to consider and dictate how long an animal should spend in a patch—the patch residency time. However, animals must not consider only the current foreground rate to optimise their foraging, they must also consider the dispersion of patches and the costs of travelling between them.

Being in a patch with a low rate of reward is sub-optimal if you could be in a different patch with a much higher rate; thus, staying until a resource is totally depleted will result in a reduced amount of reward collected in the same period of time within an environment. Residency times should therefore depend on properties of the environment outside of the current patch. In particular, it is vital to consider the cost of travelling between patches, because when travelling, the net intake of reward is zero. In addition, environments are inherently patchy and not all patches are equal, with some offering a higher yield. So, residency times should also depend on how rich an environment is, or, the average rate of rewards across all patches in the environment. Thus, optimal patch leaving decisions consider the journey that must be taken to travel between patches, the quality of the environment, as well as the rate of reward in a current patch.

How do animals make patch-leaving decisions? Krebs *et al.* (1974) established that animal behaviour could be explained by leaving patches according to a set ‘giving-up’ time, which was based on the current intake of resources in a patch, but also the environmental richness. This was subsequently formalised as an optimal foraging model called MVT (see Box 1; Charnov, 1976), which states that the optimal leaving time from any patch is when its instantaneous rate of reward equals that of the average reward rate for the environment, accounting for the costs required to move between patches. Moving earlier or later than the optimal time will lead to a reduction in the overall level of rewards collected, either through over- or under-harvesting. There is now a wealth of evidence that many species make patch-leaving decisions in line with the model, with many being very close to optimal, including guinea pigs (Cassini *et al.*, 1993), squirrels (Lewis, 1982), ladybirds (Hassell and Southwood, 1978), birds (Krebs *et al.*, 1974) and humans (Constantino and Daw, 2015; Le Heron *et al.*, 2019).

The well-studied exploration-exploitation dilemma has provided insights into how individuals choose to exploit known reward values or switch to exploring unknown alternatives, largely using forced-choice RL paradigms (Daw *et al.*, 2006). These experimental paradigms often involve learning the associations between abstract symbols and reward values. A key additional insight and contribution of MVT is that rather than learning associations, it considers the optimal duration to spend in a patch of changing value before switching to another. Furthermore, as detailed above, consideration of environmental conditions—and the cost of travelling between locations—is built into the theory and the optimal model.

Crucially, MVT is an optimal model that can be used to make specific predictions about patch-leaving times. In the next section, we outline the predicted effects MVT has on leaving times and in Box 1 we outline the model and how it can be used to predict optimal leaving times.

## Predictions of MVT

### Foreground reward rate

MVT states that the optimal time to leave a patch is when the instantaneous reward rate of the current patch is equal to the environmental reward rate. It follows therefore that a ‘high-quality’ patch with lots of resources will reach the environmental reward rate after a longer period of time. As such, MVT predicts that animals should reside in well-resourced patches longer than poorly resourced patches within the same environment. That is, you will stay in the apple tree with 20 apples longer than the one with 10 within the same environment. However, the foreground rate is not only determined by the rewards itself, but also the effort (or energetic cost) of harvesting. If collecting rewards in a patch requires a lot of energy then this effectively decreases the reward rate. So, animals will leave patches sooner if it requires a lot of effort to obtain the rewards. Thus, you will stay longer in the tree with 10 apples if they are on the lower branches than a tree with 10 apples where you have to climb to the higher branches to collect them.

### Background reward rate (or environmental richness)

Another key feature of the patch-leaving problem, compared to the type of binary choice paradigms typically used in decision neuroscience, is that the decision to leave should depend on the tracking of the average value of the environment. Within MVT, the background reward rate—or average rate of reward in all patches in the environment—also influences optimal residency times and does so independently of the foreground rate. Specifically, as shown in Box 1, it states that in rich environments where most patches are plentiful it is optimal to leave patches sooner than in poor environments where most patches are low in resources. Thus, the quality of a patch is entirely relative to the average rate of rewards of all other patches in the environment. In simple terms, a tree with 10 apples will be left sooner if the average tree in the environment has 20, than if the average tree has five.

### Travel costs

Once a decision is made to leave, you have to then travel to start collecting rewards again. But, travelling comes at a cost. While travelling the net intake of reward is zero, and thus, the length of time travelling influences the optimal time to leave. When a lengthy time must be spent travelling between patches, the environment is less rich. As a result, it is optimal to stay longer in patches in an environment where travelling takes some time. However, there is an additional travel cost, which is the amount of effort (or energy to be expended) required to get to the next patch. This has the same effect as the length of travel time, whereby higher effort costs reduce the richness of the environment and thus make it more optimal to stay in patches longer. Therefore, you will stay collecting apples from an apple tree longer if the next tree is far away or up a hill, than if it is close by on flat ground.

### Non-depleting resources

Although in most circumstances food rewards deplete within patches, there will be exceptions to this rule where resource intake does not decline over time, particularly when the resource foraged for is not a primary reinforcer. For instance, if you are in a relationship where the partner has treated you consistently well, why would you leave. MVT still makes predictions of what would happen if resource depletion is not occurring. As MVT states that the foreground reward rate is compared to the background reward rate, a decision to leave occurs when the foreground is below the background. Thus, if the foreground reward rate is above the background and does not deplete, MVT would predict an individual should stay in that patch for as long as possible. However, if a foreground reward rate does not deplete but is not above the background, MVT predicts an individual will actually leave the patch quickly, as other patches could still give a higher rate of reward. In addition, there is evidence that even if a foreground reward rate is increasing, similar neural mechanisms are used to compare that rate to the background in order to decide whether to stay or leave a patch (Wittmann *et al.*, 2016). Thus, although MVT is typically predicated on a depleting resource, its key tenets might still be useful for social neuroscience, for addressing questions such as ‘why would you leave a partner who has always treated you well?’

### Uncertainty in the MVT framework

A key assumption of MVT is complete information of the environment (Stephens and Krebs, 1986). In its original form, this assumption is reasonable, given an animal’s likely knowledge of its habitat. However, environmental richness is essentially a probability function: the animal may know the average environmental richness, but not how rich any specific patch is within it. Therefore, when choosing to leave a patch, there is no certainty that the next patch will be of high quality. Such a property of the world may also be found in social situations. For example, one may know the general reputation of a group, but not that of individual members. Still, MVT can be extended to remove this assumption. Constantino and Daw (2015) found that an MVT-informed RL model explained behaviour in a foraging task with incomplete information. Future work should examine how RL, which has increasingly been used within social neuroscience (Apps *et al.*, 2015; Lockwood and Klein-Flugge, 2019), could be integrate with MVT to better understand social decisions.

**Figure:**
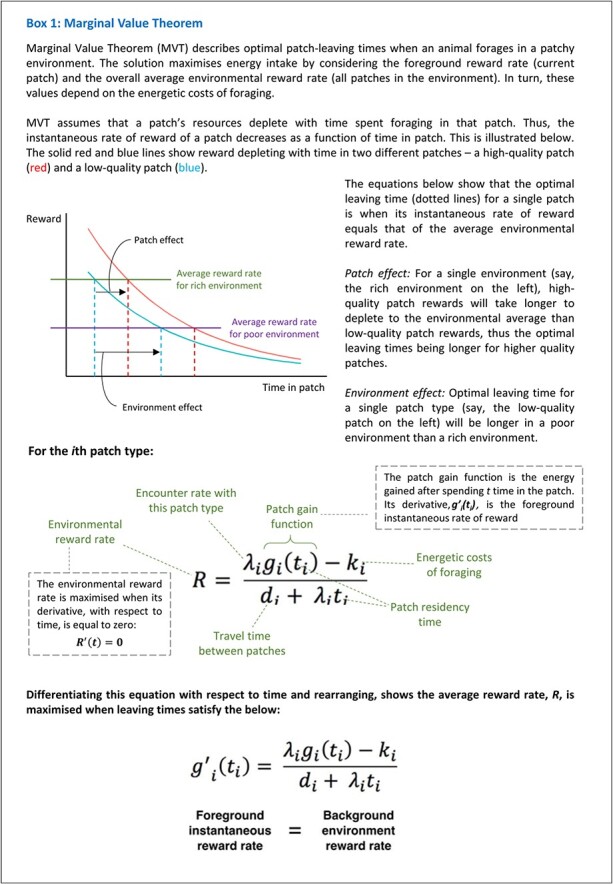
Box 1: Marginal Value Theorem

## Why forage? neural mechanisms revealed by patch-leaving paradigms

Some of the ‘decision’ variables outlined above have been examined previously in cognitive and social neuroscience, in the context of binary decisions. There is an abundance of research examining how people process temporal delays, effort costs and reward magnitudes. Many studies have looked at these in social decision-making tasks as well (Kable and Glimcher, 2007; Charlton *et al.*, 2013; Bhanji and Delgado, 2014; Ruff and Fehr, 2014; Garvert *et al.*, 2015; Apps and Ramnani, 2017; Chong *et al.*, 2017; Lockwood *et al.*, 2017; Schwenke *et al.*, 2017). However, what is unique is the ecologically valid and dynamic nature of the decision. It requires the comparison of two different reward rates, in a dynamic setting where one reward rate is constantly changing. MVT also considers efforts and delays not as purely instrumental costs that must be incurred to get a specific magnitude of reward, but instead as properties of patches and the environment that must feed into the decisions of how long to reside in a location. This is strikingly different from the two-option choice tasks that are very commonly deployed in neuroscience. Moreover, such dynamic decisions may be more reflective of many of the kinds of choices that must be made on a regular basis (Mobbs *et al.*, 2018).

### Humans conform to MVT

In addition to its potential utility in social neuroscience, there is already evidence that humans do broadly conform to MVT principles in many non-social situations. Wolfe (2013) administered a series of computerised experiments where human participants were required to forage for berries in different patches in an environment. By systematically varying different components of the task, they showed: that participants left individual patches when their instantaneous rate of reward matched the environmental reward rate; that greater travel times between patches extended residency times at individual patches; and greater within-patch ‘effort’ extended patch residency times. In these experiments, participant behaviour closely matched optimal leaving times predicted by the MVT model.

**Fig. 1:**
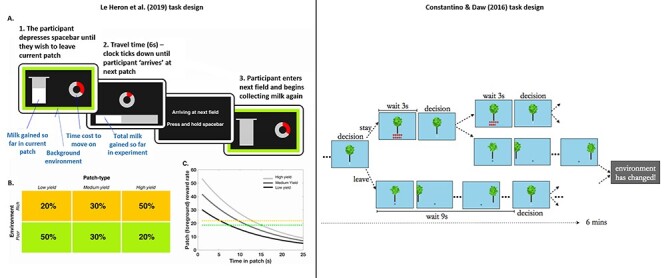
Example patch-leaving paradigms. Left panel – a continuous reward depletion design. Taken from Le Heron *et al.* (2019), (A) participants had to decide how long to remain in their current patch (field). Reward (milk) gains were indicated by the continuous filling of the silver bucket with a white bar. This gain occurred at an exponentially decreasing rate. Following a leave decision, a clock ticking down the 6-s travel time was presented, during which they could collect no reward. (B) Three foreground patch types were used, differing in the initial reward rate (low, medium and high yield). Two different background environments (farms) were used, with the background reward rate determined by the relative proportions of these patch types. Percentages represent the distribution of patch types in each environment. (C) Predictions of the optimal leaving times according to MVT. Participants should leave each patch when the instantaneous reward rate in that patch (grey lines) drops to the background environmental average (gold and green dotted lines). Therefore, people should leave sooner from all patches in rich (gold dotted line) compared to poor (green dotted line) environments, but later in high-yield compared to low-yield patches (D) In line with MVT, residency times are longer in higher yield patches, but lower in the rich environment. Right panel. Taken from Constantino and Daw (2015). (E) Participants foraged for apples in virtual patch-foraging environments. On each trial, they could choose to harvest a tree or move on to a new tree. Harvesting returned an exponentially diminishing number of apples with a short time cost. Moving to a different tree incurred a longer time cost. (F) Number of apples at last harvest is indicative of time spent in harvesting. The lower the number, the longer participants spent in harvesting each tree in that environment. Participants foraged each patch longer in richer environments (short travel times, slow decay rates)

Constantino and Daw (2015) designed an experiment where human participants foraged for apples, deciding whether to continue harvesting at a tree with depleting returns or to move on to a new, replenished tree. They found that leaving times varied as a function of environmental richness, as predicted by MVT (Figure 1). Furthermore, the authors compared how well choice behaviour fit two different learning models—a temporal difference learning algorithm or an MVT-based learning algorithm. They found that the MVT model provided a better fit to the data, suggesting an important role for environmental average reward rates in task performance. A recent study by Le Heron *et al.* (2019) used a similar design, manipulating the magnitudes of reward—or yield—available in patches and the proportion of high or low yield patches in different environments. Similarly, they found that people’s decisions, although not completely optimal, broadly conformed to the principles of MVT (Figure 1). These studies provide clear evidence of the influence of environmental context on human decisions to continue to exploit current resource options, or to move on and explore other available options.

Many human behaviours may therefore conform to MVT principles, and, as such, using these paradigms to probe human decision-making may reveal important insights into how dynamic decisions are made during social interactions.

### Novel neural mechanisms revealed by the patch-leaving problem

**Fig. 2:**
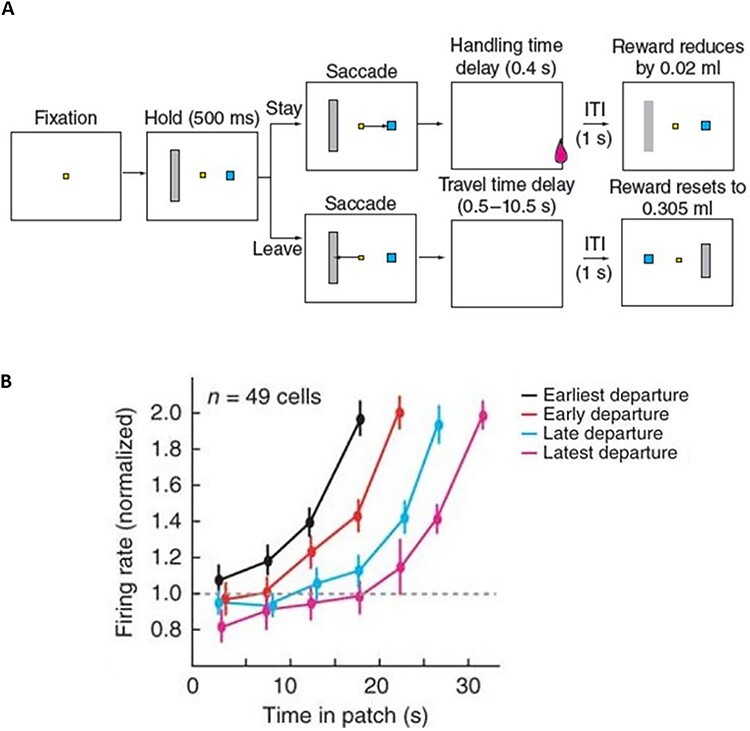
From Hayden *et al.* (2011). (A) Macaques performed a simulated patch-leaving task. On each trial, the animal could choose to stay or leave a patch by shifting their gaze to either the blue square (stay) or grey rectangle (leave). If they chose to stay, they received juice reward after a short delay, which diminished with each stay decision. If they chose to leave, they incurred a travel cost to the next patch. (B) Average firing rate of population of neurons in area 24 of the anterior cingulate sulcus. Trials split by earliness of patch leaving. Firing rates rose faster for those trials where leave decisions were made earlier, but asymptote at the same level. Error bars represent SEM.

Using MVT has already been fruitful in sub-fields of neuroscience for identifying mechanisms that may not have been revealed using other approaches. For instance, Hayden *et al.* (2011) used a patch-leaving task in macaques, recording from single-units in area 24c in the anterior cingulate sulcus (ACCs). They identified a previously never demonstrated property of cells in this region—a rise-to-threshold signal (Figure 2). The firing rate of neurons in this region increased gradually with time in a patch until the point when the monkey left a patch. Variability in leaving times correlated with variability in the rise-to-threshold, such that neurons reached a firing threshold quickest on trials with the earliest leaving times. Furthermore, when there was a greater travel time between patches, the rate of increase of firing was reduced, as well as the leaving threshold being greater. Thus, neurons in the ACCs appear to signal a rise-to-threshold signal, which builds at different rates depending on the foreground and background reward rates, but leaving occurs whenever these neurons reach the same firing rate. This work seemed therefore to have identified that this rise-to-threshold signal driving patch-leaving decisions, which would not have been identified in binary choice tasks.

In humans, Kolling *et al.* (2012) used fMRI to show that a homologous region of the dorsal anterior cingulate cortex (dACC) signals ‘search value’ during a foraging task, analogous to the average reward rate of the environment, although this was not strictly a patch-leaving task. Furthermore, they found a correlation between the variance in dACC signal and the variance in search choices across subjects. This suggests that in this context dACC signals the value of switching to an alternative option in the environment.

Patch-leaving decisions have also begun to be linked to neuromodulatory systems. Lottem *et al.* (2018) recently showed that optogenetic stimulation of serotonergic cells in the dorsal Raphe lead to greater residency times in a patch leaving task, of which some cells may in fact project to the dACC (Pollak Dorocic *et al.*, 2014). Speculatively, therefore, the serotonergic system may bias animals to stay longer in patches by modulating the threshold in the dACC for leaving. In the study, by Le Heron *et al.* (2019) they found that the delivery of a dopamine agonist to human participants performing a patch-leaving task modulated how sensitive people were to the richness of the environment, and that disruption to this dopaminergic mechanism lead to abnormal foraging behaviour in Parkinson’s disease.

The dACC is often recruited when making social decisions (Gabay *et al.*, 2014; Ruff and Fehr, 2014), and the serotonergic and dopaminergic systems have putatively distinct effects on social behaviours (Crockett *et al.*, 2015; Gabay *et al.*, 2019, 2018). Yet, the mechanisms underlying these processes have to date still not been fully understood. The evidence outlined here would therefore suggest that social neuroscience may attain significant new insight into the computations, functional anatomy and neuromodulatory mechanisms by using patch-leaving paradigms.

### Social foraging

Why might MVT be fruitful for social neuroscience? Within behavioural ecology, it is well recognised that many foraging behaviours are conducted within social settings and in social groups. One commonly used example is bees foraging for pollen. Although bees must solve the patch-leaving problem when travelling from flower to flower, such behaviour does not fit with the originally intended purpose of MVT. MVT was intended to explain how animals expend energy to obtain energy, yet for bees, the resource (pollen) does not directly contribute to an individual’s net energy intake. Instead, the pollen is a resource that is used for the benefit of the collective hive. Despite this, evidence suggests that pollinators forage in line with MVT (Cibula and Zimmerman, 1984; Goulson, 2000). This can therefore be thought of as a very simple example of a prosocial act—where the bee incurs a cost for the collective benefit. It is possible that the mechanisms that guide such ‘prosocial’ foraging behaviours may be antecedents for the altruistic behaviours of many species, including humans.

There is also evidence that many species in fact face ‘social foraging problems’, where patch-leaving decisions must be made by social groups. These decisions must still be optimised to maximise the rewards of the group and therefore conform to the principles of MVT (Rita *et al.*, 1997; Amano *et al.*, 2006). Human hunter-gatherers are a clear example of such behaviours, and there is qualitative evidence of MVT-like behaviour in hunter-gatherer foragers (Smith *et al.*, 1983). Furthermore, using historical foraging data from a nomadic hunter-gatherer society, Venkataraman *et al.* (2017), showed that the ‘residential mobility’ of a group fits well with MVT predictions. That is, the decision of when to move camp conforms with optimal leaving times based on the local habitat and broader environment in which the group resides. These observational studies point to MVT being relevant to prosocial behaviours and those requiring group-level cooperation. Experimental studies can be designed to examine the proximate, cognitive and neural mechanisms of these behaviours, testing hypotheses relating to durations of cooperative interactions, group decision-making and partner choice when making such decisions. We make recommendations as to possible task design of such experiments further below (‘Designing a patch-leaving behavioural experiment’).

Recently, behavioural and cognitive neuroscientists have begun to use patch-leaving tasks designed to examine foraging behaviour in the lab. For instance, in one study Turrin *et al.* (2017) examined how macaques forage for social information. In this study, macaques spent longer searching facial expressions when there were greater delays between conspecific encounters. Here, environmental richness was manipulated by increasing the delay between ‘patches’ and is an example of using the MVT framework in a situation where the ‘resource’ under consideration is social in nature. Behavioural data fit the predictions of MVT better than three other potential patch-leaving models. Similarly, Yoon *et al.* (2018) showed that human gaze durations are modulated by the features proposed in MVT. This suggests that animals may treat social information as a resource—or reward—to be foraged for and extends optimal foraging beyond resource collection.

In another study in humans, Zacharopoulos *et al.* (2018) required participants to make patch-leaving decisions, either to collect rewards for themselves or to collect rewards for a charity of their choice. People’s decisions were broadly in line with MVT-like predictions when collecting rewards in both conditions. Individual differences in a personally trait (personal focus) were associated with overall reward when foraging for the self, but not when foraging for charities. Such results would highlight that altruistic decisions may be influenced by some of the properties that guide patch leaving, and moreover that variability in prosocial foraging decisions may be linked to individual differences in psychiatric or personality traits.

The studies outlined above point to the potential for using this framework to understand how ‘social’ variables can be considered within a patch-leaving framework. Other social-interactive decisions may also be better understood by thinking of them as patch-leaving problems. There are instances where the value of social relationships changes over time. These include romantic relationships, platonic relationships and even business relationships (Poulin and Chan, 2010). MVT provides a novel approach from which to understand the mechanisms underlying decisions to remain in, or leave, these relationships in the face of changing value. How the value of the current social interaction may be compared to the value of other interactions, as well as the costs to switch between them, may influence the length of time one decides to remain in a relationship of depleting value.

Economic games have been used to study the contextual importance of interacting partners in terms of their fairness, cooperativeness and trustworthiness, and how this is modulated by psychiatric symptoms and pharmacological manipulation (Güth *et al.*, 1982; King-Casas *et al.*, 2005; Rilling and Sanfey, 2011; Sorgi and van ‘t Wout, 2016; Gabay *et al.*, 2019). Very little research has investigated the effect the environmental context on these behaviours. With environmental factors built into MVT computations, it provides clear opportunities to address this, providing testable hypotheses of how differences in the quality of the social environment might affect duration of social interactions.

Thus, whether using the formal framework of MVT, or simply considering social decisions as patch-leaving problems, there is potential for foraging-based frameworks to prove fruitful tools for social psychology and neuroscience.

### Foraging beyond MVT

There are other foraging models beyond MVT. These address a range of questions other than the patch-leaving problem, so are beyond the scope of this review (Mobbs *et al.*, 2015). However, these models could also be of interest for social neuroscientists to explore, so we briefly describe them here.

The diet breadth, or prey model (Stephens and Krebs, 1986), considers not durations, but whether to engage with a resource once encountered. The model formulates the optimal policy based on the net energetic gains from each resource as a function of other available resources in the environment. An important prediction of this model is that a particular resource may form an important source of food in one environment, but not be eaten at all in a richer environment (‘an ice lolly in the desert will be more valuable, and more likely to be eaten, than the same lolly in an ice cream parlour’). The same may also be true of resources, choices, in social settings. For instance, choices of social partners may depend on a subjective calculation of a person’s ‘value’ (Heijne *et al.*, 2018), and thus foraging mechanisms may also influence such choices.

The ideal free distribution model looks at the distribution of competing individuals across an environment (Fretwell, 1969; Mobbs *et al.*, 2018), and predicts that individuals distribute themselves in a manner, which minimises competition. Such a model could explain human social group behaviour, how people coordinate with others, and where people will physically situate themselves in social settings. In sum, other types of social decisions that are not about duration like in patch leaving, but are instead about whether to interact with a person or how cooperative people are, may be better understood by considering other forms of foraging paradigm.

## Designing a patch-leaving behavioural experiment

MVT is an optimal model, which allows for the designing of an experiment in which explicit and precise predictions can be made. That is, one is able to design an experiment using the MVT equation (Box 1), where the optimal leaving times can be calculated for each condition of one’s experiment. This is hugely advantageous from an experimental design perspective, but also comes with challenges that are somewhat idiosyncratic to foraging experiments. In this section, we provide practical insights in how to answer questions through experimental design in the patch-leaving framework. Furthermore, we will discuss the challenges presented by patch-leaving paradigms for probing behavioural and neural mechanisms, and potential ways to overcome them.

### Continuous versus discrete rewards

An important decision for the design of a patch-leaving task is whether the reward rate within a patch is delivered in a completely continuous manner or in a discrete ‘trial by trial’ fashion. In a continuous patch-leaving task, a participant can be shown a continuously declining reward rate (or rewards accumulating at a continuously diminishing rate) and thus is free to choose to leave at any point in time during a patch. Such a design, as deployed by Le Heron *et al.* (2019) where participants saw a milk pail continuously filling up at a declining rate, allows precise measurement of residency times relative to the foreground reward rate (see Figure 1, left panel).

Although plausible in some circumstances, there are many patch-leaving scenarios where rewards are not accrued at a continuous rate. Collecting berries or apples is not completely continuous, but can be separated into discrete moments where a reward is obtained. An alternative approach is to break the depletion up into discrete trials, where on each trial the participant must decide whether to stay or leave, with each decision to stay leading to a depleted magnitude of reward on the next trial. A decision to leave results in a period of waiting before entering a new patch that must be longer than the duration of a trial. This approach, as employed by Constantino and Daw (2015; Figure 1), solves some of the issues that arise when employing fMRI to examine neural mechanisms with a continuous version (see below). However, it offers less temporal resolution, which could potentially mask small effects on leaving times (i.e. a consistent 1 s effect on residency times in a continuous version, may not be detected in a discrete version with 3 second duration trials). A discrete version also requires modifications to the MVT equations spelt out in Box 1, although these are outlined nicely in Constantino and Daw (2015).

### Manipulating the value of patches

A cornerstone of the MVT framework is that resources within a patch deplete over time. However, there are different possible variables that can be manipulated within an experiment to create patches with different values and thus different predicted residency times. One option is to manipulate the rate of depletion, or ‘decay’ in patches; that is how quickly does the reward rate drop off. With higher depletion rates, residency times should be shorter. This manipulation was employed by Constantino and Daw (2015) with participants asked to virtually harvest apples from trees in different environments in which the apples obtained gradually declined but at different rates (see Figure 1, right panel).

An alternative method of manipulating value is to change the total reward available in patches, which effectively equates to changing the initial reward rate—or starting value. When there is more total reward available in a patch, the starting rate is higher, which leads to MVT predicting longer residency times. Such an approach was employed by Le Heron *et al.* (2019), who required participants to collect ‘milk’ from cows in fields. Those cows offered different yields of milk, which was dictated by the starting reward rate within patches.

The value of a patch can also be manipulated through the effort required to obtain a reward. When the within patch effort is higher, this creates a reduced ‘value’ patch and thus MVT predicts a reduced residency time. Although in the strictest interpretation of MVT it argues that this should be the ‘energetic’ consumed while obtaining rewards in a patch, we also know that humans value effort subjectively. As has been discussed extensively elsewhere (Westbrook *et al.*, 2013; Chong *et al.*, 2017; Kool and Botvinick, 2018), effort can be manipulated in a variety of ways by manipulating the physical or cognitive demands of a task. These manipulations may serve to change the value of patches if an effort is required to obtain a reward, even if it is not strictly an ‘energetic’ cost. To illustrate, Wolfe (2013) manipulated effort in a task requiring participants to move a cursor and click on coloured dots to obtain rewards within a square patch. Within-patch effort was manipulated by whether participants were allowed to freely move between the dots or were constrained by having to avoid green ‘bushes’, which increased the effort required to navigate between rewards in the patch.

### Manipulating the environment

Within MVT an important feature is that the effect of the different patch properties (as outlined above) should have independent effects from the properties of the environment. To identify such independent effects, it is therefore necessary to manipulate the environment in different ‘blocks’ (Constantino and Daw, 2015; Yoon *et al.*, 2018; Le Heron *et al.*, 2019). Importantly, within those blocks a variable must be manipulated for a fixed time-period, rather than a fixed number of trials—as would be typical in most cognitive experiments. This is due to the fact that (i) the number of trials a participant completes is dependent on their own leaving times and (ii) the ‘travel times’ are typically a fixed feature of an environment (Krebs *et al.*, 1974; Charnov, 1976; Stephens and Krebs, 1986). In simple terms, if you had infinite time in the experiment it would be optimal to stay in patches until all resources are depleted—but the authors are unaware of real-world environments with infinite time. Manipulations of the environment must therefore occur in distinct blocks, which are for fixed periods of time, with participant behaviour compared between environments.

If blocks differ in the effort costs of travelling, the duration of the travel time between patches, or the proportion of high yield (relative to low yield) patches within an environment, there will be a difference in the average background reward rate between blocks. As a result, MVT would predict different residency times between environments. For instance, Constantino and Daw (2015) had blocks with shorter or longer travel times between apple trees, creating ‘rich’ or ‘poor’ environments, with lower or higher residency times predicted by MVT, respectively. In contrast, Le Heron *et al.* (2019) manipulated the environment by having blocks with a majority of ‘high yield’ patches or blocks with a majority of low yield patches, with MVT predicting shorter or longer residency times, respectively. Thus, these variables that manipulate the properties of the environment have been found to manipulate human patch leaving times in line with MVT.

As noted above, patch leaving requires participants to make decisions of a different nature (‘stay or leave’) than typical binary choice paradigms. But, can patch-leaving decisions be explained by pre-existing computational models? This is an important question when designing a patch-leaving experiment, and there have been different approaches to showing that pre-existing RL-based models do not predict MVT-based behaviours. One approach has been to instruct participants that there are different types of blocks, but that they must learn each block’s properties by sampling patches. Constantino and Daw (2015) showed that even when participants are required to learn the properties of the environment in this manner, they make decisions that conform to MVT’s principles and that the MVT model is a better fit to the data than a commonly deployed RL-based model. Similarly, Hayden *et al.* (2011) showed that a temporal discounting model could not account for the decisions of macaques in a patch-leaving foraging task. In addition, it has also been suggested that drift-diffusion models can be adapted to accommodate MVT variables to better characterise patch-leaving choices, and their underlying mechanisms (Davidson and Hady, 2019).

An alternative approach has been to control for any possibility of learning, by explicitly instructing and training participants about the properties of the different environments (Le Heron *et al.*, 2019) (i.e. instruct participants that in a particular block of the experiment there is a higher proportion of high yield patches). When doing so, there is no learning during the experiment that would fit with an RL-based account.

### Practical challenges

There are inherent challenges when designing patch-leaving experiments, due to the nature of the decisions being made and due to previously observed human biases. In most decision-making paradigms, a participant will make a choice on every trial between two options. The experimenter therefore has complete control over the number of data-points (decisions) in each condition within the experimental design. In a patch-leaving paradigm where blocks have fixed time periods, but patch residency times depend on the participant, it is impossible to completely pre-determine how many times you will sample each patch type in each environment. This is because the participant may choose, for example, to stay at each high-quality patch for a long time, thus reducing the available time to see low-quality patches. As a result, most patch-leaving paradigms will result in unbalanced designs and different numbers of samples per participant. In addition, although one of the advantages of the optimal MVT framework is the potential for knowing precisely how long residency times should be given the properties of the experiment, humans show a bias that needs to be taken into consideration. In several experiments, it has been shown that humans tend to stay in all patches longer than is optimal, in a manner that is invariant to the properties of the experiment (Constantino and Daw, 2015; Le Heron *et al.*, 2019). Thus, it is likely that an experiment will end up in fewer samples than would be predicted by the optimal model.

How can such challenges be overcome? Firstly, careful piloting of the task is required to ensure the length of time in each environment is sufficient to obtain enough repeats of each type of patch to enable robust statistical analysis. Secondly, the unbalanced nature of the design calls for the usage of mixed effects models that are robust to variability in the number of samples from each participant (Bates *et al.*, 2015). Thirdly, make predictions about the number of samples that will be obtained based on the MVT model, but account for the additional staying bias. Thus, make blocks longer—or include more repetitions of blocks—to obtain sufficient samples.

## Examining the neural mechanisms of patch leaving

One of the major advantages of the patch-leaving framework is that decisions are not made between binary options, but instead one must constantly compare an evolving foreground reward rate with the alternative ‘background’. Such an evolving comparison occurs over a longer timescale (e.g. ~15 s) in most experiments, allowing one to start examining the mechanisms that guide decisions about rewards (or social variables) that one typically cannot easily measure in commonly employed fMRI designs. For instance, as noted above Hayden *et al.* (2011) identified a ‘rise-to-threshold’ signal in the dACC during patch leaving. Although this was in single neurons, the nature of patch-leaving decisions means that putatively similar mechanisms may be examined in human brains using fMRI. Normally this would not be possible due to the sluggish nature of the BOLD signal and the rapid nature of most decisions in binary choice paradigms. In order to take advantage of this aspect of patch-leaving paradigms, however, there are still practical challenges to be overcome.

### Going beyond standard block or event-related fMRI analyses

fMRI paradigms are often either ‘block’ or ‘event-related’ in nature. In both such cases, ‘subtraction’ designs are typically used whereby one compares the BOLD signal evoked by one condition against that of a second condition (Amaro and Barker, 2006; Penny *et al.*, 2011). Although such approaches can be employed to examine aspects of patch leaving, they will miss the rich nature of the signals that can be identified during foraging tasks, such as rise-to-threshold signals and thus the key computations involved in patch-leaving decisions.

To illustrate the challenge, let us assume an experiment where two environments differ only by travel time between patches (long or short). In such an experiment, the relevant difference is the environmental reward rate, which would be lower in the long travel time compared to the short. If one simply defined the blocks of interest as being the whole period one was in an environment, the contrast would reflect differences in activity related to environmental richness. While this could provide fruitful information, it would not tell you how activity evolved differently between the patches. Such a contrast would entirely miss the continuous extended comparison between foreground and background reward rates. As such a challenge that must be solved is how to analyse patch-leaving data, when standard fMRI analysis tools are not sufficient.

There are a few solutions to this problem, some based purely around statistical analysis approaches, and others solve the problem by tweaking the designs of patch-leaving tasks. One simple solution is to use a patch-leaving task where the foreground reward rate is not continuously delivered, but instead happens with discrete ‘events’. For instance, in Constantino and Daw (2015), different magnitudes of apples are delivered every few seconds. One can therefore treat this as an event-related design, and use parametric modulators to examine activity time-locked to these events that scales with some features of your patch-leaving experiment. For instance one could include parametric modulators incorporating the difference between current patch reward and the environmental average. This would capture a dynamic comparison and would allow regions that were involved in computing such an evolving decision variable to be revealed.

It is important to note, which when using parametric modulators to analyse an event-related design, assumptions are made about the nature of the BOLD signal. Specifically, one assumes some form to the haemodynamic response following an event (e.g. a standard assumption in fMRI packages is that the evoked response peaks at 6 s after stimulus and returns fully to baseline at 32 s). However, when examining a rise-to-threshold signal, it is not clear whether a smooth signal that builds up gradually within a patch (Figure 2) should conform to such assumptions. Although such assumptions are often made across a range of different fMRI analyses approaches, it is relevant to note this as a caveat here. In the future, it may therefore be fruitful to use the parametric-fMRI-based approaches suggested here, in parallel with imaging methods that have better temporal resolution such as EEG or MEG, to better understand how such an evolving decision variable relates to neural mechanisms.

In designs where the foreground reward rate continuously depletes, using a parametric modulator in the typical way may not be ideal for the nature of the experiment. In continuous designs, one does not have an event to ‘time-lock’ the analysis to, and as a result, it is unclear what an ‘event’ should be. How can one analyse an fMRI study that does not have standard events? McGuire and Kable (McGuire and Kable, 2015) provide an elegant solution to such a problem. They analysed fMRI data from a paradigm that although not a patch-leaving experiment, had many similarities. In the paradigm participants had to decide how long to persevere as reward value changed. Such a design created data of a form similar to that which would be generated in a patch-leaving paradigm.

To analyse their data, instead of modelling individual events and fitting the assumed canonical haemodynamic response function—that peaks at 6 s and returns to baseline at 32 s—they used finite impulse response to model the BOLD signal (Henson *et al.*, 2001). This FIR analysis approach was applied across the whole time-period where participants were evaluating whether to persevere or not as a reward value continuously changed. The FIR approach consists of defining post-stimulus time-bins; for example, every 2 s from entering a patch. Each time-bin is included as a regressor in a first-level analysis, and the resulting betas represent the signal change of each time-bin from baseline. By comparing the same time bins between different types of patch or different environments it would be possible, as shown in Mcguire and Kable (2015), to show the BOLD signal changing dynamically whilst participants are in patches. Thus, although not ‘events’ per se, the FIR approach allows one to look at how the BOLD signal changes over a period of time, which is ideal for patch-leaving decisions. Relying on ‘modelled’ betas from a FIR analysis may not even be necessary. It is also possible to simply extract BOLD signals from regions of interest for a period of time leading up to a decision to leave, or from the time-point of entering a patch, and examining how different features of the experiment influence these raw BOLD signals. This may initially seem complicated, however, this is no different from how data are extracted to make peri-stimulus time histogram plots in many fMRI papers. Moreover, the betas from the FIR time bins or time-bins of extracted BOLD signals can be analysed in the same way that any other time-series data are. As a result, standard univariate or multivariate analysis techniques can be used to examine how the BOLD signal develops leading up to patch-leaving decisions in relatively straightforward, albeit atypical, ways.

### De-correlating predictors

**Fig. 3:**
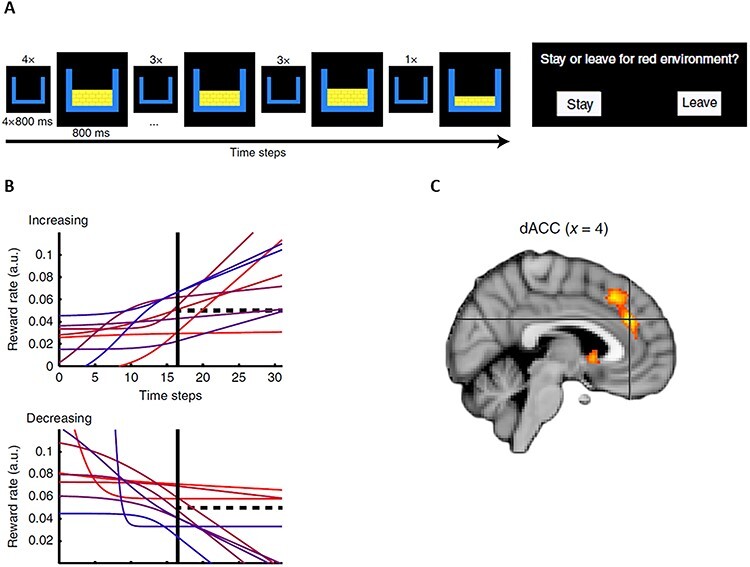
From Wittmann *et al.* (2016). (A) Trial structure: participants saw a series of ‘reward events’. These gave rewards of different levels, including no reward. Participants could infer the reward rate by the interval between rewarded events and the magnitude of the rewards. After a fixed period, they chose between staying in the current patch or moving to a previously learned default patch. (B) Trials were derived from 18 different reward curves, nine of which increased, nine of which decreased, monotonically. This approach allowed for decorrelation between the foreground reward rates in different patches. The decision time is indicated by the solid black line, and the default reward rate is indicated by the dotted black line. (C) The dACC showed greater activity with a greater positive reward trend.

An important consideration in all computational fMRI studies is to make sure that one’s predictors or regressors time-locked to events in the experiment are not too strongly correlated. This is particularly pertinent in patch-leaving tasks, because foreground reward rates always deplete, and thus can make highly correlated predictions about the BOLD response. One solution to the problem is to make sure at the design stage that the depletion rates are very distinct, to ensure low correlations. However, this may not always be possible, given the other constraints on patch-leaving tasks. An alternative solution is to slightly modify a task, such as in the manner employed by Wittmann *et al.* (2016). They used a design with a fixed decision point in every patch, after a set number of rewarded timepoints (Figure 3). At this decision point, participants chose whether to stay or switch to a default patch (which can be thought of as the background reward rate), whose reward rate had been learned during task-training. To decorrelate foreground reward rates, they used multiple distinct trajectories to the accrual of rewards, with rates monotonically increasing or decreasing during each patch. So, although these were not strictly the depleting rewards as prescribed by MVT, the need to track the foreground reward rate (or trajectory) and compare it to an alternative has considerable similarities. In this way, they were able to examine which brain regions tracked the increasing or decreasing reward trend. This design provides an elegant approach to decorrelating different foreground reward rates; moreover, it again highlighted the dACC as a key region for making a comparison between different rates of reward.

## Conclusion

Here we highlight the potential for using a hugely influential model from behavioural ecology, MVT, to probe the neural mechanisms underlying social behaviours. This ‘patch-leaving’ framework outlines the importance of the richness of the environment, and the quality of one’s current location, as key drivers of a dynamic decision-making process. We have shown that many species, including humans conform to its principles and that many social behaviours may necessitate making decisions about how long to stay in a patch. By outlining the promise and pitfalls of designing patch-leaving experiments to probe behaviour and its underlying mechanisms, we hope to encourage the wider usage of foraging-based approaches in social neuroscience.
